# Percutaneous transhepatic coil embolisation of a common hepatic artery aneurysm in vascular Ehlers–Danlos syndrome

**DOI:** 10.1186/s42155-025-00600-8

**Published:** 2025-10-16

**Authors:** Oliver Chan, Hariesha Pathmaraj, Chris Grieco, Nadeem Shaida

**Affiliations:** 1 University of Cambridge School of Clinical Medicine, Hills Road, Cambridge, CB2 0SP UK; 2 Department of Radiology, Addenbrooke’s Hospital, Hills Road, Cambridge, CB2 0QQ UK

**Keywords:** Vascular Ehlers–Danlos syndrome, Aneurysm, Common hepatic artery, Percutaneous transhepatic, Coil embolisation, Interventional radiology

## Abstract

**Background:**

Vascular Ehlers–Danlos syndrome is a rare connective tissue disorder characterised by arterial fragility, predisposing patients to life-threatening vascular complications. Endovascular aneurysm management in these individuals poses significant challenges due to their delicate vasculature and limited surgical options. This case report highlights the novel use of a direct percutaneous transhepatic approach for aneurysm coiling in a patient with a rapidly expanding hepatic artery aneurysm, demonstrating an innovative solution to a complex vascular emergency.

**Case presentation:**

A 17-year-old male with a known diagnosis of vascular Ehlers–Danlos syndrome presented with a perforated sigmoid colon. After undergoing a midline exploratory laparotomy, imaging revealed a rapidly expanding 50-mm aneurysm in his common hepatic artery. Traditional endovascular coiling was infeasible due to significant proximal stenosis, creating similar limitations for vascular reconstruction and liver transplantation. The multidisciplinary team opted for a direct transhepatic approach to coil the aneurysm. The patient recovered without complications, and follow-up imaging confirmed haemodynamic stability and adequate liver perfusion.

**Conclusions:**

This case highlights the importance of an integrative multidisciplinary approach in managing complex vascular emergencies and successfully demonstrates how a direct percutaneous transhepatic approach can serve as a valuable reference for similar cases, expanding the repertoire of endovascular interventional radiology techniques for challenging pathologies.

## Background

Vascular Ehlers–Danlos syndrome (vEDS) is a rare, autosomal dominant connective tissue disorder caused by mutations in the COL3A1 gene, leading to defective type III collagen synthesis [1]. This defect causes extreme fragility of arterial walls and hollow organs, such as the bowel and uterus [1, 2]. Patients can present with life-threatening complications, including arterial rupture, aneurysms, or spontaneous bowel perforations [3]. This condition is estimated to affect 1 in 50,000 to 1 in 200,000 births [2]. While there is no cure, management focuses on regular surveillance, supportive care, and mitigating lifestyle and surgical risks [1]. For women with vEDS, genetic counselling and pregnancy risk assessment are essential components of care.

## Case presentation

A 17-year-old male presented to Addenbrooke’s Hospital emergency department with sudden onset lower abdominal pain radiating to his groin. His mother brought him in, reporting concerns about a possible aortic dissection, which she had experienced in the past. Both the mother and son were known to have vEDS.

The remainder of the patient’s history, physical examination, bedside investigations, and blood tests was unremarkable. Abdominal computed tomography (CT) imaging revealed scattered locules of intraperitoneal free gas and fat stranding around a loop of bowel in the left iliac fossa, suggesting a sigmoid colon perforation.

The patient underwent an exploratory midline laparotomy and Hartmann’s procedure to repair the perforation and form a stoma. Due to no identifiable secondary cause, his perforation was presumed to be a spontaneous rupture secondary to vEDS.

**Fig. 1:**
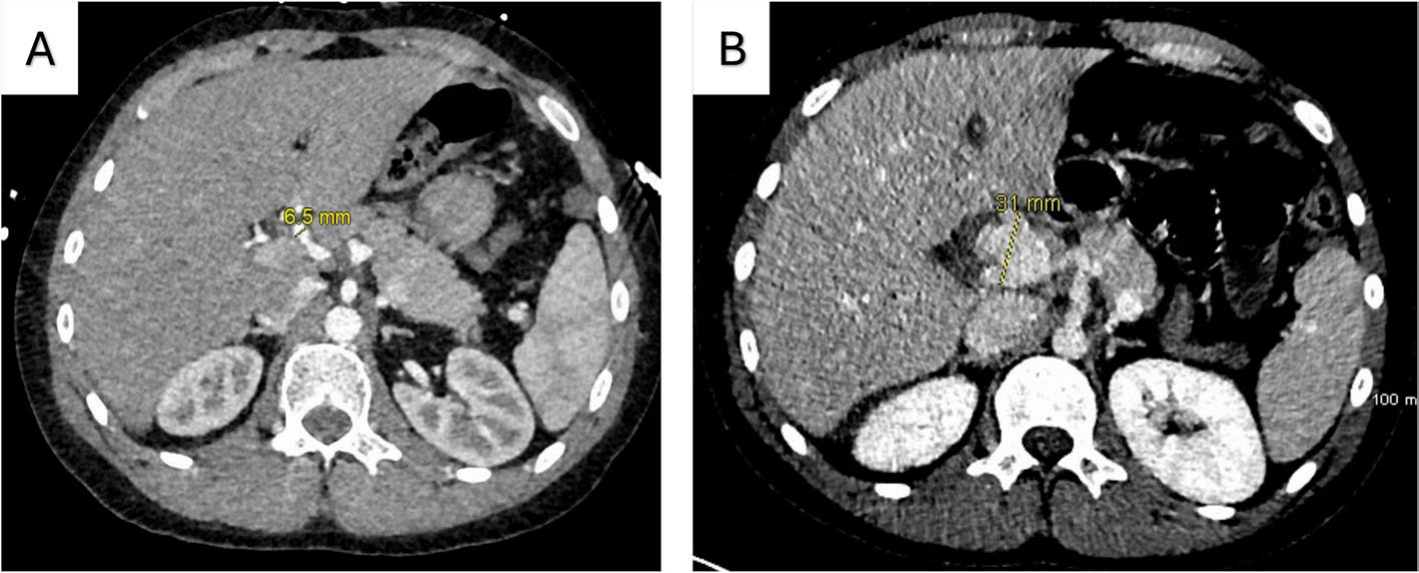
Comparison of pre-laparotomy (**A**) and post-laparotomy CT scans (**B**). **A** Pre-laparotomy CT shows the common HA at 6.5 mm. **B** Common HA dilated to 31 mm in 5 days

A postoperative CT on July 4 (day 5 post-op) confirmed the successful repair of his colon perforation but failed to identify a developing aneurysm in his common hepatic artery (HA). Retrospective review revealed that the common HA had expanded from 6.5 mm on June 29 (pre-op) to 31 mm, indicating rapid growth over 5 days (Fig. 1A, B).

After initial recovery, the patient was discharged on July 10 (day 11 post-op). However, he was readmitted the next day due to poor wound healing and right upper quadrant pain. A repeat CT scan on July 16 (day 17 post-op) successfully identified the aneurysm, which had now rapidly grown to 50 mm. His left HA had also dilated to 18 mm, escalating the situation to a vascular emergency due to imminent risk of rupture.

### Differential diagnoses

The severity of the gut perforation and vascular instability observed in this case aligns with the patient’s underlying vEDS, as intestinal and arterial fragility are hallmark diagnostic criteria for this condition. These manifestations may occur spontaneously, independently of external insult.

One potential consideration is that the common HA aneurysm was exacerbated by the surgical intervention. For example, incidental trauma or haemodynamic stress during his laparotomy could have contributed to arterial wall weakening. However, this explanation is unlikely, as the surgical site lies anatomically distant from the common HA and no intraoperative vascular injury was documented.

Another possibility is that the common HA aneurysm developed secondary to colonic perforation, resulting in a mycotic aneurysm. However, the rapid expansion over a 5-day period is inconsistent with typical mycotic aneurysm progression. Furthermore, there was no evidence of bacteraemia [4], and the concurrent dilation of the left HA suggests a broader vasculopathic process rather than a localised infectious cause.

### Management options

Given the complexity of this case, a multidisciplinary team of interventional radiologists, hepatobiliary, paediatric transplant, and vascular surgeons was needed to assess the viability of different management options.

Endovascular coiling, a minimally invasive technique performed by interventional radiologists, is commonly used to provide haemodynamic stability in aneurysms [5, 6]. However, in this case, severe stenosis of the proximal common HA increased the risk of arteriography-related complications, which are already significant with vEDS [7, 8]. An alternative approach via the superior mesenteric artery, pancreaticoduodenal arteries, and then the gastroduodenal artery was considered but faced the same limitations.

A second option was liver transplantation combined with vascular surgery. While this approach was potentially curative for both the aneurysm and the dilated left HA, transplantation requires reconstructing the common HA, which was excessively stenosed [9]. Additionally, the on-call vascular surgery team did not have extensive subspecialty experience in connective tissue disease vascular surgery, leading to the dismissal of this option even before evaluating this patient’s transplant candidacy and associated challenges.

The third option was a direct transhepatic puncture of the common HA. Hepatobiliary and interventional radiology teams determined that the aneurysm could be directly accessed through the abdominal wall under ultrasound guidance. This had the advantage of bypassing complex vasculature but carried significant risks such as damage to surrounding structures and catastrophic internal bleeding. This was a novel approach, as primary interventions typically involve open [10], endovascular [11], or hybrid techniques [12]. Reports of aneurysm management with direct percutaneous access are extremely limited in this population. After weighing all the available options, the team decided that this approach was the best course of action.

### Outcome

**Fig. 2:**
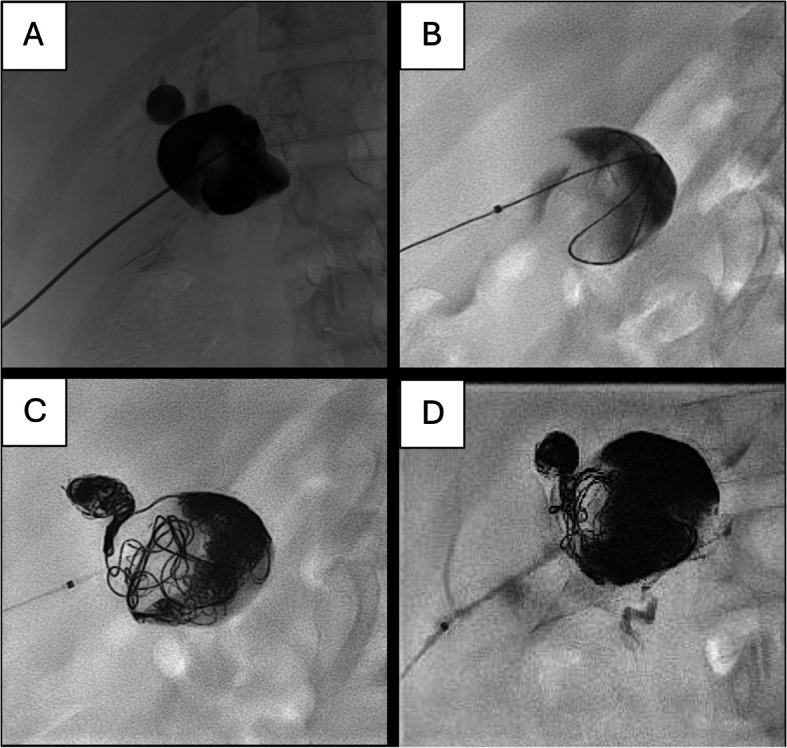
Direct transhepatic puncture into the common HA aneurysm. **A** Anteroposterior fluoroscopy showing 6 Fr sheath transhepatic access. **B** Coiling of large common HA aneurysm. **C** Aneurysmal left HA cannulated and embolised with coils. **D** Embolisation with tantalum liquid embolic (Onyx) showing opacification of gastroduodenal artery inferiorly

The interventional radiology team successfully coiled both the common HA aneurysm and the dilated left HA (Fig. 2A, B, C, D) using a 6 French gauge (Fr) sheath to deliver 27 microcoils. Ultrasound and fluoroscopy were used to guide direct transhepatic access, with a curvilinear probe employed for real-time ultrasound guidance. Arteries supplying the aneurysm, such as the gastroduodenal artery, were embolised using a total of 12 vials of Onyx. The tract was embolised with Spongostan (gelfoam) upon sheath withdrawal to minimise bleeding risk.

Several periprocedural precautions were taken given the high risk of vascular injury. While under general anaesthesia, a consultant anaesthetist inserted a central venous catheter into the right internal jugular vein and placed a radial arterial line. Informed consent was obtained from the patient and family for transfusion of blood products, potential conversion to open surgery, and the risk of catastrophic fatal bleeding. In anticipation of possible bleeding complications, the haematology team was consulted about the perioperative use of tranexamic acid, desmopressin, and vitamin K.

Post-procedure, following 6 h of bed rest, Doppler ultrasound confirmed that the aneurysm was no longer filling, and the patient’s liver remained adequately perfused through collateral circulation. The patient stabilised without complications and was discharged after 9 days, with follow-up surveillance via a combination of ultrasound and abdominal and pelvic magnetic resonance angiography to monitor disease.

While this approach proved successful, it is important to acknowledge that direct transhepatic aneurysm coiling may not be feasible in all centres. It is operator dependent, carries significant inherent risks, and requires multidisciplinary planning and a high level of expertise in interventional radiology.

## Conclusions

1. vEDS has a complex disease pathogenesis which requires close surveillance and supportive management.
2. Embolisation techniques like vascular coiling are effective radiological interventions to manage vascular emergencies.
3. An integrative, multidisciplinary approach is important to manage challenging pathologies, and cooperation in specialised input leads to improved patient outcomes.
4. Due to the complex pathology of vEDS, critical signs can be overlooked by seasoned radiologists if they are not specifically searched for.
5. Direct transhepatic puncture of the HA aneurysm, while successful in this instance, is operator dependent, carries significant risks, and may not be suitable in all centres without specialised expertise.

## Data Availability

All data generated or analysed during this study are included in this published article and its supplementary information files.

## Abbreviations

vEDS: Vascular Ehlers–Danlos syndrome
CT: Computed tomography
HA: Hepatic artery
Fr: French gauge
